# Protocol for the methamphetamine approach-avoidance training (MAAT) trial, a randomised controlled trial of personalised approach bias modification for methamphetamine use disorder

**DOI:** 10.1186/s13063-020-04927-6

**Published:** 2021-01-06

**Authors:** Joshua B. B. Garfield, Hugh Piercy, Shalini Arunogiri, Dan I. Lubman, Samuel C. Campbell, Paul G. Sanfilippo, Jeff Gavin, Malcolm Hopwood, Eli Kotler, Suzanne George, Goke Okedara, Lara R. Piccoli, Victoria Manning

**Affiliations:** 1 Monash Addiction Research Centre, Eastern Health Clinical School, Monash University, Melbourne, Australia; 2 Turning Point, Eastern Health, 110 Church Street, Richmond, Melbourne, Victoria 3121 Australia; 3 Association of Participating Service Users (APSU), Self Help Addiction Resource Centre (SHARC), 140 Grange Road, Carnegie, Melbourne, Victoria 3163 Australia; 4 Department of Psychiatry, University of Melbourne, Melbourne, Australia; 5 Albert Road Clinic, Ramsay Health, 31-33 Albert Road, Melbourne, Victoria 3004 Australia; 6 Malvern Private Hospital, 5 Wilton Vale Crescent, Malvern East, Melbourne, Victoria 3145 Australia; 7 Monash Alfred Psychiatry Research Centre, Monash University, Melbourne, Australia; 8 School of Psychological Sciences, Monash University, Melbourne, Australia

**Keywords:** Methamphetamine use disorder, Approach bias, Cognitive bias modification, Addiction, Rehabilitation, Relapse

## Abstract

**Background:**

Globally, methamphetamine use has increased in prevalence in recent years. In Australia, there has been a dramatic increase in numbers of people seeking treatment, including residential rehabilitation, for methamphetamine use disorder (MUD). While residential rehabilitation is more effective for MUD than withdrawal treatment (i.e. “detoxification”) alone, relapse rates remain high, with approximately half of rehabilitation clients using methamphetamine within 3 months of rehabilitation. “Approach bias modification” (ABM) is a computerised cognitive training approach that aims to dampen automatically triggered impulses to approach drugs and drug-related stimuli. ABM has been demonstrated to reduce alcohol relapse rates, but no randomised controlled trials of ABM for MUD have yet been conducted. We aim to test whether a novel “personalised” form of ABM, delivered during rehabilitation, reduces post-treatment methamphetamine use, relative to a sham-training control condition. Secondary outcomes will include dependence symptoms, cravings, and approach bias.

**Methods:**

We aim to recruit 100 participants attending residential rehabilitation for MUD at 3 sites in the Melbourne metropolitan area. Participants will complete baseline measures of methamphetamine use, craving, dependence severity, and approach bias before being randomised to receiving 6 sessions of ABM or “sham” training. In the active condition, ABM will be personalised for each participant, using those methamphetamine images that they rate as most relevant to their recent methods of methamphetamine use as “avoidance” images and using positive images representing their goals or healthy sources of pleasure as “approach” images. Approach bias and craving will be re-assessed following completion of training, and methamphetamine use, dependence, and craving will be assessed 4 weeks and 3 months following discharge from residential treatment.

**Discussion:**

This study is the first randomised controlled trial of ABM for MUD and also the first ABM study to test using a personalised set of both approach and avoid images for ABM training. If effective, the low cost and easy implementation of ABM means it could be widely implemented as a standard part of MUD treatment.

**Trial registration:**

Australian New Zealand Clinical Trials Registry ACTRN12620000072910. Registered on 30 January 2020 (prospectively registered): https://www.anzctr.org.au/Trial/Registration/TrialReview.aspx?id=378804&isReview=true

## Background

Globally, the use of amphetamines—particularly methamphetamine—has grown significantly in recent years. The estimated number of people who used methamphetamine in the past year rose from 24 to 34.2 million between 2006 and 2017 [1, 2]. In Australia, while the percentage of the population reporting past-year use of illicit amphetamines appears to have declined over this period [3], shifts among people currently using methamphetamine towards using more potent forms of methamphetamine (i.e. “ice” instead of “speed”), more frequent use, and more rapid-onset routes of administration (i.e. smoking and injecting instead of snorting or swallowing) have led to an increased number of people experiencing methamphetamine use disorder (MUD) [4]. Amphetamines are now the second-most common primary drug of concern (PDOC), after alcohol, among clients of Australian substance use treatment services, accounting for 27% of all treatment episodes [5]. Among clients of residential rehabilitation (the most intensive form of substance use treatment), amphetamines are now the most common PDOC.

While residential rehabilitation, particularly with longer duration of treatment, is a more effective treatment for MUD than withdrawal treatment (i.e. “detoxification”) alone [6], relapse remains common after rehabilitation. Australia’s first methamphetamine treatment outcome study, “MATES”, found that 47% of clients used methamphetamine within 3 months, and 78% within 1 year, after commencing residential rehabilitation [7]. Given that residential rehabilitation is a highly cost-intensive and scarce resource, it is critical that post-rehabilitation outcomes are optimised. Novel neurocognitive interventions show promise as adjunctive interventions that can be easily added to existing withdrawal, rehabilitation, and counselling programmes.

According to contemporary neurocognitive models of addiction, substance use disorders are maintained, at least in part, by a bias towards a relatively automatic, impulsive style of information-processing and decision-making in response to drug-related thoughts and stimuli [8]. This bias reduces the influence of the more reflective style of processing/decision-making that involves consideration of long-term goals and values and can lead to the inhibition of initial impulses [9, 10]. According to these models, information processing involves a series of iterations, beginning with predominantly limbic and striatal processing responsible for activating initial approach/avoidance tendencies [11]. If response selection requires a more reflective, instead of automatic, processing style, subsequent iterations of processing may then engage fronto-cortical regions, allowing associations and contextual information to be activated to guide response.

However, repeated use of addictive drugs can cause neuroadaptations in the limbic system which sensitise early, relatively automatic attentional and behavioural reactions. This is reflected in *attentional bias*, the tendency for drug-related cues in the environment to selectively capture attention, and *approach bias*, automatically activated action tendencies to approach drugs due to their attributed motivational significance [12–14]. People with problematic alcohol, nicotine, cannabis, and heroin use demonstrate an approach bias to drug cues [15], and some studies find approach bias to be associated with increased substance use, problem severity, and craving [16, 17]. Approach bias is automatically activated before reflective processing can be engaged. Moreover, in people with MUD, cortical deficits and accompanying cognitive impairments, including impaired attention, executive functions, working memory, and inhibitory control, are common [18, 19] and may make it difficult to engage reflective processes that could inhibit drug approach impulses. Theoretically, this combination of overactive approach bias and underactive reflective processes may cause behaviour to become increasingly influenced by drug-related cues in the environment [20].

Rehabilitation programs often include psychotherapeutic interventions to improve clients’ abilities to identify triggers, control cravings, and strengthen planning skills to avoid drug use and reorient towards healthier goals. These approaches aim to improve “reflective” aspects of cognition known as “executive control,” which is involved in evaluating information, choosing courses of action, and inhibiting maladaptive responses. However, even if executive control is strengthened by psychotherapeutic interventions, less-conscious cognitive biases may remain present and re-emerge and influence behaviour if executive control is later weakened (e.g. due to stress). The average duration of methamphetamine use for people accessing residential rehabilitation for MUD is 12 years [7], and cognitive biases that can “trigger” renewed drug cravings and drug-seeking are therefore likely to be deeply ingrained. This highlights the importance of interventions that directly target cognitive biases. Perhaps the most promising such intervention is approach bias modification (ABM).

The first ABM training task targeting a substance use disorder was developed for alcohol use disorder (AUD) [21]. In typical alcohol ABM tasks, participants are presented with alcohol-related pictures to which they repeatedly make an “avoidance” movement (e.g. using a joystick to “shrink” or “push away” an alcohol-related picture presented on a computer screen) and neutral (non-alcohol) stimuli to which they repeatedly make an approach movement. This allows individuals to practise overriding their automatic approach tendency by repeatedly performing an avoidance response, such that avoidance becomes more automatic, thereby reducing or inhibiting approach bias. In AUD samples, 4–12 sessions of ABM reduces approach bias [14, 21–24] and reduces neural signatures of alcohol cue reactivity in the amygdala [25] and medial prefrontal cortex [23]. Importantly, when delivered during residential withdrawal or rehabilitation treatment, ABM training reduces rates of post-treatment alcohol relapse [14, 22, 24, 26, 27]. Indeed, ABM has now been included in German national guidelines for the treatment of AUD [28].

ABM has recently been trialled as a treatment for tobacco use in several small trials [29–31], with one study reporting that it significantly reduced nicotine consumption 3 months after training, relative to a sham training control condition [30]. A small proof-of-principle study has also found that 4 sessions of ABM reduced cue-induced craving in cannabis users [32]. Aside from our single-group, open-label feasibility pilot study [33], there have been no trials of ABM for methamphetamine use to date.

Our recent pilot study tested ABM in 47 MUD clients undergoing 1–2-week residential withdrawal treatment (i.e. “detoxification”). ABM was well-tolerated, despite the fact that it involves exposing participants to potentially triggering drug-related images. Only 4 participants (9%) withdrew due to finding the training distressing, and in post-training ratings of the task, 78% “agreed” or “strongly agreed” that the task was interesting. Among participants who completed a 3-month follow-up, the past-month abstinence rate was 54%, which compares favourably to the rate of past-month abstinence reported at the 3-month follow-up in detoxification clients in the MATES study (18%) [7]. However, a high proportion of our participants were lost to follow-up, suggesting we may have overestimated the actual abstinence rate, and there was no control group to compare the abstinence rate to. Moreover, the proportion of participants who completed the full 4-session protocol (62%) was slightly lower than observed with an alcohol withdrawal sample [24, 26]. Nevertheless, these data indicate good acceptability, moderate feasibility, and a possible clinical benefit of ABM. We anticipate higher acceptability and intervention completion rates in rehabilitation clients, who are likely to be more clinically stable than acute withdrawal patients.

In alcohol ABM research, and in our pilot methamphetamine ABM feasibility study, training programs have used a standard picture set for all participants, regardless of which specific forms of the substance they consume or which routes of administration they use. However, our experience conducting research in AUD patients suggests that participants often drink only one type of beverage, or only a small range of beverages, while our pilot study in MUD patients suggested that most participants use only one method of administering methamphetamine (i.e. only smoking or only injecting) [33]. Since approach bias is the product of repeated associative conditioning experiences, it is likely to be specific to stimuli resembling the drug types and paraphernalia frequently used by an individual. Thus, in protocols that use a standardised set of images intended to represent a wide range of drug forms and paraphernalia, many images used are likely to have little relevance to most individuals (e.g. repetitive responding to beer images may have limited effect for someone who only drinks wine; images of a glass pipe used for smoking methamphetamine may elicit relatively little approach bias for someone who only injects methamphetamine). Using more “personalised” picture sets for ABM training may therefore increase its “potency” for reducing approach bias, and therefore relapse rates.

Thus, for the first time in any ABM study, we will trial an ABM programme in which the drug-related stimuli are “personalised”, i.e. where each participant will be trained using only those images they rate as being most personally relevant. We will test whether this approach results in superior treatment outcomes relative to a “sham training” control condition that is modelled on sham training conditions that have been used in alcohol ABM research, i.e. using a standard picture set for all participants and using training instructions that do not systematically train them to either approach or avoid either methamphetamine or non-methamphetamine images. This will also be the first trial in a clinical population that aims to train participants to approach positive stimuli aligned with their goals and values, in the hopes that this will reinforce these goals at a subconscious level, strengthening treatment outcomes further.

## Methods

### Aims

Our primary objective is to test whether 6 sessions of “personalised” ABM, relative to a “sham training” control condition, increases the likelihood of past-month abstinence from methamphetamine 1 month and 3 months after discharge from rehabilitation. As a secondary measure of efficacy, we will also test whether ABM increases the rate of continuous abstinence over the whole 3-month follow-up period.

Other secondary objectives include:

1. Testing whether ABM reduces methamphetamine approach bias, relative to sham training.
2. Testing whether ABM reduces methamphetamine craving, relative to sham training.
3. Testing whether ABM reduces severity of MUD after discharge from rehabilitation, relative to sham training.
4. Testing whether ABM delays the time to first lapse to methamphetamine use after discharge from rehabilitation, relative to sham training.
5. Testing whether dimensions of impulsivity, particularly positive and negative urgency, correlate with methamphetamine approach bias, and whether they moderate the effect of ABM on methamphetamine use, dependence symptoms, and approach bias.
6. Measuring the acceptability of this novel ABM training task.

### Trial design

This is a randomised, double-blind, sham-controlled, parallel group trial. The protocol has been formulated in accordance with Good Clinical Practice, SPIRIT, and CONSORT 2013 guidelines.

### Study setting

We will conduct this trial at 3 services which offer 4-week residential addiction rehabilitation programmes. In these programmes, clients’ withdrawal symptoms are medically managed (typically during the first week of withdrawal) and clients participate in a range of group therapy activities. One site is a public substance use withdrawal management and stabilisation service attached to a large public hospital. Another site is a private psychiatric hospital which offers an addiction treatment programme. The third site is a standalone private addiction rehabilitation hospital. All sites are located in the Melbourne metropolitan area, Australia.

### Sample size

We aim to recruit 100 participants. This trial is partly intended to establish effect size estimates for future larger trials, and the projected sample size for this trial is not based on an a priori power calculation. This will be the first randomised controlled trial (RCT) of ABM in a MUD sample; hence, there is little basis to estimate an expected effect size. The sample size was therefore pragmatically based on an estimate of the number of participants that could realistically be recruited from the participating sites within the available recruitment period. Across the 3 recruitment sites, an average of approximately 9 MUD clients are admitted each week. In our pilot study [33], we found that approximately two thirds of MUD clients met eligibility criteria, and approximately half of those who were eligible (i.e. approximately one third of all MUD admissions) agreed to participate. As such, we anticipate that we will recruit an average of 3 participants per week across the 3 sites, allowing a sample of 100 to be recruited in approximately 8 months. To ensure that opportunities for recruitment are not missed, the project manager liaises with intake staff on a weekly basis in order to check that they have screened every admission for eligibility, so that all clients entering treatment at the study sites and meeting eligibility criteria can be approached for recruitment.

### Eligibility criteria

Participants must be aged at least 18 years; meet at least 4 DSM-5 criteria for MUD within the past 3 months; have used methamphetamine on at least 4 of the 28 days prior to commencing residential treatment; have sufficient English language proficiency to understand the participant information sheet, questionnaires, and intervention task instructions; have a phone number which we can use to contact them for follow-up interviews; and be intending to stay in residential treatment long enough to complete the 6-session protocol. Patients are excluded from participating if the clinicians who assess them at admission judge them to be too physically or mentally impaired to provide informed consent or to safely participate in the study.

### Measures

#### Demographic and clinical characteristics

A researcher administers a structured questionnaire assessing basic demographics (e.g. age, gender, etc.) and clinical characteristics (e.g. previous residential drug treatment, other drugs of concern, self-reported history of psychiatric diagnoses).

#### MUD severity

The MUD module of the Structured Clinical Interview for DSM-5 Disorders—Research Version [34] (SCID-5-RV) is administered to confirm eligibility and measure severity of MUD. The time-frame of the SCID questions is altered from the standard “past year” to “the 3 months prior to starting treatment” (at baseline) and “past 3 months” (at the 3-month follow-up), so that equivalent periods of time are being assessed at each time point. The Severity of Dependence Scale (SDS) [35] is used to provide a continuous measure of psychological dependence on methamphetamine. At baseline, the timeframe that the SDS items addresses is “the month prior to entering residential treatment” and at follow-ups the timeframe is “the past month”. At follow-ups, if the participant reports no methamphetamine use in the past month, the SDS is not administered and they are automatically assigned a score of zero.

#### Substance use

A timeline follow-back (TLFB) interview [36] is used to confirm eligibility and measure frequency and quantity of methamphetamine use and route of administration. To aid description of the sample, information regarding tobacco, alcohol, and other illicit/non-prescribed substance use is also recorded using the TLFB. Time frames covered by the TLFB are as follows: the 28 days prior to commencing residential treatment (at baseline), the 28 days following discharge from residential treatment (1-month follow-up), and the past 28 days (3-month follow-up).

#### Methamphetamine craving

The Craving Experience Questionnaire (CEQ) [37] is used to measure frequency of methamphetamine craving in the past week and strength of the strongest craving experienced in the past week. The CEQ consists of two 10-item scales, one measuring the frequency of cravings over a defined time period (in this study, the past week), and the other assessing the strength of the strongest cravings during this period. Each item is rated on a scale from 0 to 10. Each 10-item scale can further be broken down into 3 factors: “intensity”, “imagery”, and “intrusiveness”. While the CEQ has not been validated specifically for methamphetamine, its factor structure has been validated for both alcohol and tobacco and over a range of time frames (including the past week).

#### Impulsivity

The 20-item short version of the UPPS-P Impulsive Behavior Scale (S-UPPS-P) [38] will be used to assess five distinct traits related to impulsivity: sensation seeking, premeditation (lack of), perseverance (lack of), negative urgency, and positive urgency. This instrument contains five subscales representing these five traits. Each subscale is composed of four items rated on a 4-point Likert scale ranging from “strongly agree” to “strongly disagree” [38]. This short version has shown good internal consistency (Cronbach’s alpha = 0.74–0.88 across the five subscales) and is a reliable and valid alternative to the full UPPS-P [38].

#### Picture selection

To identify the 10 most personally relevant pictures in each image category (methamphetamine and positive) for each participant, participants are asked to view 50 methamphetamine-related images (e.g. pictures of ice and speed, and paraphernalia such as glass pipes and injection equipment) which include the 40 images that can be used in the ABM training and 10 additional images only used in approach bias assessment (details below). Using a laptop, participants rate each image on a computerised visual analogue scale (scored 0–100) in response to the question: “How much does this image remind you of the times you’ve used methamphetamine”. They are also asked to view the 50 positive images (e.g. images representing family or friends enjoying time together; financial success; employment; exercise, sports, and recreational activities; healthy foods; pets; travel and holidays, etc.) and asked to rate each one in response to the question, “How much does this image remind you of your goals, things you enjoy, or things you would like to spend more time doing?”. Participant responses are written to local storage as a JSON-formatted text file, comprising an array of *name*: *value* pairs (specifically, the image name and its associated rating). The methamphetamine-related and positive image sets were selected in consultation with a focus group comprised of people with a lived experience of treatment for methamphetamine use disorder, which was conducted as a preliminary project (Monash University Human Research Ethics Committee project number 21625).

#### Approach bias

Participants complete a computerised approach-avoidance task (AAT) to measure approach bias. We use a relevant-feature version of the task as this may achieve a more reliable measurement of approach bias than irrelevant-feature tasks. As described by Kersbergen, Woud, and Field [39], this is the only version of the AAT that has thus far been shown to have acceptable internal consistency (Cronbach’s alpha = .70 in an alcohol version of the task). Thus, participants are presented with 2 blocks of 80 randomised images (the same 20 methamphetamine-related and 20 positive images in each block, each presented twice in each block) on a laptop computer screen. For both classes of images, 10 are a set of pictures (same for all participants) used only in the AAT. The remaining 10 are the most highly rated of the remaining 40 pictures that are also used in the ABM training.

In the first block, participants are instructed to push a joystick in response to methamphetamine-related pictures and pull the joystick in response to positive images. In the second, these instructions are reversed, so that participants are required to pull the joystick in response to methamphetamine-related images and push in response to positive images. Pushing the joystick causes the image to shrink until it disappears, creating the impression of the image “receding” into the “distance”, as if pushing the joystick has actually “pushed” the image “away”. Pulling the joystick causes the image to increase in size until it is double the height and width of its original display (i.e. filling as much of the screen as possible, while still displaying the whole image) when the joystick is pulled to the maximum extent, creating the impression of “approach”, as if pulling the joystick has “pulled” the image “towards” the participant. Thus, for each image included in the AAT, participants perform an “approach” action twice in one block and an “avoidance” action twice in the other block.

Participants are instructed to try to respond as quickly and accurately as possible. Each block begins with 8 practise trials (4 methamphetamine-related, 4 positive, all chosen from the set of images only used in the AAT) to ensure participants understand the task instructions and to help stabilise reaction time (RT). RT is not recorded for practise trials, but only for the subsequent 80 trials in each block. During both practise and measurement trials, if participants make an error by moving the joystick to the maximum extent in the wrong direction, a red “X” will appear on the screen and participants are required to repeat the trial. RTs from error trials or from “second attempts” after an error are not recorded. Approach bias scores are calculated by subtracting mean reaction times on approach trials from mean reaction times on avoidance trials according to the method outlined by Kersbergen, Woud, and Field [39]. Approach bias scores are also calculated separately for the 10 personalised images from each category (to test approach bias to the most relevant stimuli) and the 10 generic images from each category only used in the AAT (to allow us to test whether changes in approach bias following ABM training generalise to images not used in training).

#### Task acceptability

Participants are asked to self-administer the Endorsement and Discomfort Scale (EDS) [40] to rate the acceptability of the ABM training. Following the scoring method described by Milosevic, Levy, Alcolado, and Radomsky [41], a single score is calculated for the whole scale. The wording of the instructions and the questions addressing each item were adapted for this study based on advice from Irena Milosevic (personal communication), the lead author of the manuscript which describes this scoring method.

### Intervention

Both the sham and ABM conditions involve 240 trials per session. We use an irrelevant-feature version of the modified AAT [14] (i.e. implicit training) which helps maintain participant blinding. Images are presented surrounded by a rectangular “frame” (i.e. a black rectangle surrounding the image) which is in either “portrait” or “landscape” orientation. Both conditions involve the same task instructions: participants are instructed to “push” the joystick in response to pictures with a landscape-oriented frame and “pull” in response to pictures with a portrait-oriented frame. Push and pull responses cause the image to shrink and expand, respectively, as previously detailed in the description of the AAT.

In the sham condition, 80 pictures (40 methamphetamine and 40 positive; i.e. all pictures except the 20 used solely for the AAT) are each presented in random order, three times per session. In the ABM condition, only the 10 of the 40 ABM pictures from each category that were most highly rated in the picture selection task (i.e. 20 pictures in total) are used, each presented 12 times. In the sham condition, methamphetamine-related images and positive images each comprise 50% of portrait-oriented images and 50% of landscape-oriented images, so that participants are not systematically trained to approach or avoid either type of image. In the ABM condition, 95% of the “push away” (i.e. landscape-oriented) trials are methamphetamine-related (and 5% positive) and 95% of the “pull” (portrait-oriented) trials are positive (and 5% methamphetamine-related), to systematically train participants to avoid methamphetamine-related images and approach positive images. The inclusion of 5% of trials where the content-orientation correspondence is reversed is intended to ensure that participants attend to task instructions (i.e. to make response decisions based on picture orientation, rather than content) and to improve the likelihood of participants being blind to condition in case participants from different conditions discuss the study with each other.

In both conditions, each session is preceded by a display of the task instructions, followed by 8 practise trials in which participants will respond to empty rectangles (4 in landscape and 4 in portrait orientation), to ensure they are familiar with the task instructions before commencing the training trials. During both practise and training trials, if participants make an incorrect response (i.e. move the joystick to its maximum extent in the incorrect direction), a red “X” is displayed and they are required to repeat that trial. The training task takes around 15 min to complete.

### Allocation

Randomisation occurs automatically at the time participants commence their first session of ABM. A randomisation sequence detailing which participant numbers are assigned to which condition is programmed into each laptop computer, using a blocked 1:1 allocation ratio. Since separate laptops are used for each recruitment site, randomisation is therefore stratified by site. Randomisation sequences were generated by the computer programmer responsible for programming the ABM tasks prior to commencing the study. The randomisation sequences are stored within a database on a password-protected server. The program’s access to these data is automated and manual access to the database is limited to the programmer, chief investigator, and a statistician not involved in data collection. A local copy of the randomisation sequence stored on the computer is encrypted to prevent accidental unblinding (i.e. to prevent staff involved in recruitment at sites from knowing what condition a participant will be assigned to prior to them commencing the first ABM session, and to prevent staff involved in conducting follow-ups from becoming unblinded to participant condition prior to assessing outcomes). When participants commence an ABM session, the researcher enters the participant’s number, and the ABM program queries the database (or the local copy, if there is no internet connection) to determine the condition associated with that number.

### Procedure

**Fig. 1:**
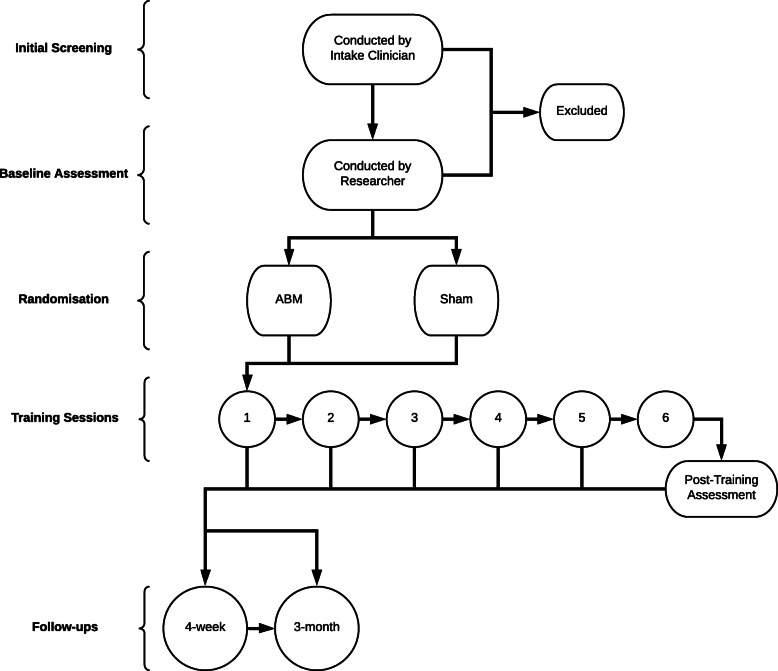
Participant flow through stages of the study

Clinicians responsible for admitting patients to the sites screen clients within the first week of their admission to rehabilitation. If a client is deemed eligible, the clinician informs them of the opportunity to participate in research and asks them if they are interested in discussing it with research staff. If the patient expresses interest, the clinician alerts the research team, and a research assistant then approaches the patient (approximately 1 week after admission to residential treatment) to provide a verbal and written explanation of the project and collect written informed consent if the client is willing to participate. A master version of the participant information sheet and consent form can be found on the Australian New Zealand Clinical Trials Registry (https://www.anzctr.org.au/Trial/Registration/TrialReview.aspx?id=378804&isReview=true) (Fig. 1).

Following this, the researcher administers the interviewer-administered questionnaires (demographic/clinical history, SCID, TLFB). The researcher also collects contact details that can be used to contact the participant once they have discharged from residential treatment, including phone numbers, email and postal addresses, messaging app details, and contact details of others (e.g. friends, relatives, or clinicians) who can help pass messages onto the participant if we are otherwise unable to contact them. The participant then self-administers the SDS and CEQ. This is followed by the picture selection task and AAT, and then the first session of ABM. Due to the length of the recruitment process, questionnaires, and tests administered prior to commencing the first session, there is flexibility regarding whether these procedures are all done on the same day, or split into 2 separate sessions (e.g. baseline questionnaires in one session and session 1 in a separate later session, as shown in Table 1). Moreover, the first session of ABM is conducted at least 7 days after the participant commences residential treatment, to minimise the degree to which acute withdrawal symptoms (e.g. fatigue, emotional lability) interfere with engagement in the training task.

**Table 1:** Schedule of measures and interventions

Expected times:	*1 week after admission to rehabilitation*	*1–3 weeks after admission to rehabilitation*			*4 weeks after leaving rehabilitation*	*12 weeks after leaving rehabilitation*
Time point:	*Baseline*	*Session 1*	*Sessions 2–5*	*Session 6*	*4-week follow-up*	*3-month follow-up*
*Eligibility screening*	X					
**Assessments:**						
*Demographic and clinical history*	X					
*SCID-5-RV*	X					X
*SDS*	X				X	X
*TLFB*	X				X	X
*CEQ*		X		X	X	X
*Picture selection*		X				
*AAT*		X		X		
*EDS*				X		
*Blinding check*						X
**Intervention:**						
*ABM—training*		X	X	X		

*AAT* approach-avoidance task, *ABM* approach bias modification, *CEQ* Craving Experience Questionnaire, *EDS* Endorsement and Discomfort Scale, *SCID-5-RV* Structured Clinical Interview for DSM-5 Disorders—Research Version, *SDS* Severity of Dependence Scale, *TLFB* timeline follow-back

ABM is repeated 3 times per week for 2 weeks, for a total of 6 sessions. There is flexibility regarding frequency of sessions to try to ensure that as many participants as possible complete 6 sessions prior to discharge. For example, if a session is missed due to participant illness, additional sessions can be scheduled after 2 weeks if 6 have not yet been completed at that point, or more than 3 sessions can be conducted in a week if the participant is not staying longer (or if their planned discharge is moved to an earlier date). However, only one session can be administered per day (i.e. multiple training sessions are not conducted on the same day).

Immediately before and after each ABM session, participants are asked to rate the intensity of their current methamphetamine craving on a single-item visual analogue scale (VAS). The VAS is presented with the question, “How strong is your craving for methamphetamine right now” displayed above it, and is anchored with the words “not at all” and “extreme” at the left and right ends of the scale, respectively. Ratings are recorded on a 0–100 scale. If a participant rates their craving in the right half of the VAS (i.e. closer to “extreme” than “not at all”) at any time, the researcher administering training checks if the participant feels comfortable continuing to participate, reminds them that they can withdraw at any time, and checks if they would like support from the rehabilitation facility’s clinical staff. The same steps are taken if a participant expressed distress at any time during their participation while they are staying at the rehabilitation facility. If they request support from clinical staff, the researcher will cease any study procedures and find a member of clinical staff. The intervention will be discontinued if the participant expresses a desire to withdraw from the research or if clinical staff at the recruitment site inform the researchers that the participant is no longer able to participate. If any participant expresses distress during their participation, this is reported to the trial coordinator to be recorded, regardless of whether or not it results in the participant withdrawing from the trial.

Following the final session of training, the AAT and CEQ are re-administered. Participants are also asked to complete the EDS to assess task acceptability. Participants are given a $60 supermarket gift card for completing training. Following their discharge from the site, a researcher obtains (de-identified) information from the site clinical records regarding the participant’s history of physical health and psychiatric diagnoses, substance use history, and medications administered to them during their residential stay.

One and 3 months following discharge from rehabilitation, a researcher not involved in administering training (and therefore blind to treatment condition) phones the participant and administers the TLFB, CEQ, and SDS. At the 3-month follow-up, the SCID-5-RV is also administered. We also assess continuous abstinence since discharge with a categorical (yes/no) question regarding any illicit amphetamine use since the previous follow-up and, if any has been used, the date of first use is assessed to allow survival analysis of time to first lapse. One-month follow-ups will occur no earlier than 28 days after discharge, and may be conducted up to 56 days post-discharge. Three-month follow-ups may be conducted no earlier than 84 days (i.e. 12 weeks) post-discharge and may be conducted up to 150 days post-discharge. Participants are sent a $20 supermarket gift card for each follow-up completed. At the 3-month follow-up, we also ask participants “Do you believe you received the form of training intended to reduce impulses to approach drugs or do you believe you were in the placebo group who received a form of training that was not designed to reduce approach impulses?” to assess blinding.

In accordance with intention-to-treat principles, we attempt to follow up all participants who are randomised (i.e. who commence the first session of ABM training), regardless of how many sessions they complete, unless they withdraw from participation. If a participant tells a researcher that they wish to withdraw (or if they tell clinical staff at the residential facility, and this is communicated to a member of research staff) the researcher seeks to ascertain their reason for withdrawal and communicates this to the study coordinator, so that the reason for withdrawal can be recorded. Reasons for withdrawal are classified according to whether they are related to the acceptability of the study protocol (e.g. finding the ABM training or questionnaires distressing) or are unrelated (e.g. the participant simply losing interest in participating, or facing stressors unrelated to study participation).

### Data management

Questionnaires are computerised and administered, using Qualtrics software, on the laptops used for training (except for the TLFB, as described below). Paper copies are available in case an internet connection is unavailable, and if questionnaire data is collected in this way, the researcher who collected the data inputs it into the Qualtrics database at the soonest available opportunity. The TLFB is administered on paper, and the variables to be derived from it are then entered into Qualtrics by researchers experienced in conducting and interpreting TLFB interviews. All TLFB data are subject to double-entry (i.e. entered by two separate researchers into 2 separate Qualtrics pages). At the conclusion of the study, these two entries will be examined for discrepancies, which will be corrected to produce a final version of the methamphetamine use data. Upon completion of the computerised tasks (i.e. picture selection, AAT, and ABM), response/performance data files will be sent to the trial coordinator for storage on a shared drive in a folder accessible only to the researchers involved in this study. The researcher on site will also save a local text file on the laptop used to administer these measures/tasks.

All Qualtrics entries, paper questionnaires, and computer task data files are labelled only with the participant’s number, not their name or other identifying details. The only form on which the participant number is listed alongside their name and contact details is the locator form, which is stored in a locked filing cabinet separate to where other paper data is stored, and the computer file containing these details is protected with a unique password shared only with the chief investigator and those staff responsible for conducting follow-ups. Once data collection and cleaning is complete, all data stored on Qualtrics will be downloaded into files to be stored in the project folder on a password protected drive and will be deleted from Qualtrics. The final dataset will be under the custodianship of the chief investigator, and access to it is limited to the investigators and trial staff approved by the primary ethics committee to be part of this trial.

### Outcomes and statistical methods

#### Primary outcome

The primary outcome is past-month abstinence from methamphetamine, as measured by the TLFB at each follow-up. The 3-month follow-up is the primary end-point for this outcome, and the 1-month follow-up will also be analysed as a secondary end-point. At each end-point, this outcome will be tested by comparing the proportion of participants reporting past-month abstinence using Pearson’s chi-square tests.

#### Secondary outcomes

In addition to testing past-month abstinence as the primary outcome, we will also test a more stringent operationalisation of abstinence—continuous abstinence since discharge, as measured at the 3-month follow-up—as a secondary outcome. Comparison of groups, in terms of proportion of participants reporting continuous abstinence, will be analysed using a Pearson’s chi-square test. Approach bias will be analysed using mean AAT score, with the end-point being after session 6 of ABM. Change in AAT score between baseline and session 6 will be analysed using a linear mixed-effect model (LMM) testing the main effects of group and time, and their interaction.

Methamphetamine craving will be analysed using mean scores on the frequency and strength scales of the CEQ, with end-points including post-training (session 6), 1-month, and 3-month follow-ups. Two separate LMMs (one for the CEQ frequency scale, and one for the CEQ strength scale) will be used to analyse reduction in scores at these end-points, relative to score at baseline. Both LMMs will test the effect of group, time (4 levels: baseline, post-training, 1-month follow-up, and 3-month follow-up), and the interaction between these 2 effects.

MUD severity will be indexed using mean scores on 2 different measure—the SDS and SCID-5-RV. End-points for this outcome are the 1-month follow-up (SDS only) and at the 3-month follow-up (both SDS and SCID-5-RV). Separate LMMs for each measure will be used to test change in score, relative to baseline. Both LMMs will test the effect of group, time, and their interaction, although for SDS, time will have 3 levels (baseline, 1-month follow-up, 3-month follow-up), while for SCID-5-RV, it will only have 2 levels (baseline, 3-month follow-up).

For MUD severity, binary categories will also be derived from each of the measures used. For SCID-5-RV, participants will be classified according to whether they meet MUD criteria at the 3-month follow-up. For SDS, participants will be classified according to whether they have a score of 5 or more at each end-point. For each of these measures, proportions of participants in each category will be compared between groups using Pearson’s chi-square. Time to first lapse (i.e. number of days between discharge and first methamphetamine use, measured using a single question incorporated into the TLFB interview at both follow-ups) will be compared between groups using Cox regression analysis. Acceptability of the intervention following the final session of ABM will be assessed using EDS descriptive statistics (mean, standard deviation).

Pearson’s correlation tests will be used to examine the association between impulsivity and approach bias. Separate logistic regression models, testing each dimension of impulsivity, along with group and the interaction term between group and the impulsivity dimension, will be used to explore which dimensions of impulsivity moderate the effectiveness of ABM on abstinence from methamphetamine. LMM will be used to explore whether any dimensions of impulsivity moderate the effect of ABM on continuous outcomes.

The primary analyses will be conducted on an intention-to-treat basis, whereby attempts will be made to follow-up everyone who commences a single session of training. Missing outcomes (i.e. from those lost to follow-up) will not be imputed in the primary analysis, though supplementary sensitivity analyses of past-month and continuous abstinence will be conducted in which we will assume all those lost to follow-up used methamphetamine. Additional “per-protocol” supportive analyses will be restricted to participants who completed at least 4 full sessions of training, and to those who completed all 6 sessions.

All statistical analyses will be conducted by an unblinded statistician who is not involved in participant recruitment or data collection. Interim analyses conducted or supervised by the unblinded statistician are permitted for the purpose of any reports (e.g. student projects, reports to the funding body) that are due prior to completion of the project. These analyses will be accessible to the chief investigator and any other relevant personnel (e.g. the student and their supervisor, in the case of a student report). The trial will end when we have recruited 100 participants—there is no intention to stop the trial in response to interim analyses.

### Dissemination plan

We intend to present the findings of this study at the National Centre for Clinical Research on Emerging Drugs (NCCRED) symposium as part of the annual Australasian Professional Society on Alcohol and Drugs (APSAD) conference. We also intend to publish the outcomes in peer-reviewed scientific journals. Publications of trial results will be conducted solely by the investigator team with no professional or external writers to be used. Any investigator or project personnel who has had a significant role in developing the protocol, analysing data, and writing the manuscript will have the right to authorship of the manuscript reporting the primary outcomes, with chief investigator VM to determine order of authorship. Authorship of any manuscripts reporting additional secondary analyses will be determined by VM depending on individuals’ contributions to the specific aspect of the study being reported. A lay summary of the findings will also be made publicly available on the Turning Point website (www.turningpoint.org.au).

## Discussion

This will be the first RCT of ABM for methamphetamine use or indeed (to our knowledge) for any stimulant use disorder. The majority of people with MUD are likely to have lapses to methamphetamine use, even after relatively intensive treatments such as residential rehabilitation [7]. Finding ways to augment treatment success rates therefore remains important, particularly given the recent rises in numbers of people with MUD [4], increased MUD presentations to addiction treatment services [5], and the significant health, social, and economic burdens associated with methamphetamine use [42, 43].

ABM is worthy of trialling for MUD for several reasons. Firstly, we have already conducted a feasibility pilot study in which we found it was acceptable to most MUD patients who participated [33]. Although feasibility (in terms of rate of recruitment, and rate of completion of all sessions) was limited in this study, this was due less to the nature of the training task itself and more to the difficulties of conducting that study in an acute withdrawal setting (typically 7–10 days residential treatment). Specifically, withdrawal-related fatigue, and a high rate of unexpected early discharges from residential treatment meant many patients could not be recruited and many participants were unable to complete all ABM sessions. We anticipate that the 4-week treatment programmes where we are conducting the current trial will improve rates of recruitment and retention, since the flexibility afforded by the longer residential treatment duration means we can approach and train participants after the most debilitating period of early withdrawal has passed. Moreover, despite the difficulties we faced retaining participants in the feasibility pilot study, the surprisingly high rate of abstinence from methamphetamine we observed at the 3-month follow-up in that study gives us optimism that we may find ABM to be effective when directly compared with a sham-training control condition.

This is also the first clinical trial of ABM to use personalised approach and avoid stimuli. Despite numerous recommendations that approach stimuli should be personalised to align with patients’ goals for behavioural change and alternative strategies to deal with stress (e.g. personal health, reconnecting with family and friends, exercise) [44–47], only one small trial testing ABM for tobacco smokers has used personalised approach imagery [46]. Moreover, the use of personalised drug-related images to increase the efficacy of ABM by tailoring stimuli towards individuals’ preferred drug forms and routes of administration has not yet been tested. Following preliminary evidence that ABM can be used to simultaneously reduce approach bias to an unhealthy behaviour (alcohol use) and increase approach bias towards a healthy behaviour (condom use) [48], we anticipate that personalising both approach and avoid stimuli may help participants abstain from methamphetamine and also engage in activities aligned with their treatment goals. It should be noted however that the present study will not compare personalised ABM against standard ABM (which uses a generic set of images for all participants) due to recruitment constraints, although this will be important to examine in a well-powered head-to-head trial in the future.

An additional strength of the present study is that, prior to finalising the protocol, we consulted a focus group comprising people with lived experience of treatment for MUD who provided extensive advice on the drug and positive picture sets, as well as advice on recruitment materials and computerised task instructions. We expect this will also improve the acceptability of this trial. This trial will also include several outcome measures (abstinence, craving, and dependence symptoms), consistent with recommendations from a panel of treatment and research experts convened by the US National Institute on Drug Abuse (NIDA) [49].

Several practical and logistical issues may pose potential challenges to the timely completion of this study. Firstly, the acute symptoms of methamphetamine withdrawal (e.g. extreme fatigue, emotional lability) and unexpected changes in participant discharge dates may hinder participant retention and completion of the full study protocol. It is also possible that these issues may contribute to a poor follow-up rate (as observed in our initial pilot trial, where only 55% of participants completed the 3-month follow-up) [33]. However, unlike our pilot trial which was conducted during the first 7–10 days of methamphetamine withdrawal, the present trial will allow sessions to be conducted up to 28 days after cessation of methamphetamine use, and it is therefore expected that participants will be more clinically stable and more able to comply with the study protocol. It should also be noted that due to the potentially triggering nature of the methamphetamine-related images and assessments, participants’ cravings will be measured before and after each training session, and those in need of additional support will be referred to clinical staff at the rehabilitation service. Additionally, details of a 24/7 alcohol and other drug helpline will be offered to all participants during the 1- and 3-month follow-up interviews.

Despite these concerns, we anticipate that ABM holds great promise for the treatment of MUD. ABM is a low-cost intervention, requiring no specialist skills to administer, and can be delivered across a variety of settings (e.g. inpatient and outpatient treatment, home-based delivery, etc.). If proven effective, its low cost and easy implementation means ABM could address a significant gap in the treatment of MUD and help to curtail the increasing harms associated with this chronic, relapsing disorder.

## Trial status

This trial protocol is currently version 2, dated February 17, 2020. Recruitment for this trial commenced on March 16, 2020, and we anticipate recruitment will be completed in August, 2021.

## Data Availability

De-identified participant data (both questionnaire data and computer task performance data) may be made available to other researchers upon reasonable request to the chief investigator, and only following further approval from the Eastern Health Human Research Ethics Committee. Researchers wishing to access individual-level data will need to contact the chief investigator, Victoria Manning (victoria.manning@monash.edu).
